# Targets and Mechanisms Associated with Protection from Severe Plasmodium falciparum Malaria in Kenyan Children

**DOI:** 10.1128/IAI.01120-15

**Published:** 2016-03-24

**Authors:** Linda M. Murungi, Klara Sondén, David Llewellyn, Josea Rono, Fatuma Guleid, Andrew R. Williams, Edna Ogada, Amos Thairu, Anna Färnert, Kevin Marsh, Simon J. Draper, Faith H. A. Osier

**Affiliations:** a KEMRI Wellcome Trust Research Programme, Centre for Geographical Medicine Research-Coast, Kilifi, Kenya; b Unit of Infectious Diseases, Department of Medicine, Solna, Karolinska Institutet, Stockholm, Sweden; c The Jenner Institute, University of Oxford, Oxford, United Kingdom; d Nuffield Department of Medicine, Centre for Clinical Vaccinology and Tropical Medicine, University of Oxford, Churchill Hospital, Oxford, United Kingdom

## Abstract

Severe malaria (SM) is a life-threatening complication of infection with Plasmodium falciparum. Epidemiological observations have long indicated that immunity against SM is acquired relatively rapidly, but prospective studies to investigate its immunological basis are logistically challenging and have rarely been undertaken. We investigated the merozoite targets and antibody-mediated mechanisms associated with protection against SM in Kenyan children aged 0 to 2 years. We designed a unique prospective matched case-control study of well-characterized SM clinical phenotypes nested within a longitudinal birth cohort of children (*n* = 5,949) monitored over the first 2 years of life. We quantified immunological parameters in sera collected before the SM event in cases and their individually matched controls to evaluate the prospective odds of developing SM in the first 2 years of life. Anti-AMA1 antibodies were associated with a significant reduction in the odds of developing SM (odds ratio [OR] = 0.37; 95% confidence interval [CI] = 0.15 to 0.90; *P* = 0.029) after adjustment for responses to all other merozoite antigens tested, while those against MSP-2, MSP-3, Plasmodium falciparum Rh2 [*Pf*Rh2], MSP-1_19_, and the infected red blood cell surface antigens were not. The combined ability of total IgG to inhibit parasite growth and mediate the release of reactive oxygen species from neutrophils was associated with a marked reduction in the odds of developing SM (OR = 0.07; 95% CI = 0.006 to 0.82; *P* = 0.03). Assays of these two functional mechanisms were poorly correlated (Spearman rank correlation coefficient [*r_s_*] = 0.12; *P* = 0.07). Our data provide epidemiological evidence that multiple antibody-dependent mechanisms contribute to protective immunity via distinct targets whose identification could accelerate the development of vaccines to protect against SM.

## INTRODUCTION

Severe malaria (SM) afflicts young children, commonly under the age of 5 years, in areas with stable and high malaria transmission intensity (1–3). Children present at the hospital with three main and often overlapping syndromes of coma (cerebral malaria), severe anemia (hemoglobin [Hb] < 5 g/dl), and respiratory distress, alongside other complications, such as convulsions and hypoglycemia (4). For every 200 children infected with Plasmodium falciparum, it is estimated that at least 2 will develop SM and that 1 of them will die (5). The global mortality attributable to malaria in 2013 was approximately 855,000, the majority of whom were African children (6). Furthermore, among survivors, severe and long-term neurological impairments are frequent even when appropriate treatment is received (7).

Why some P. falciparum infections are asymptomatic while others result in a severe and life-threatening illness that may cause lifelong disability has long been debated and, remarkably, still remains unclear (5, 8, 9). The reasons are thought to be multifactorial, including host genetic factors, age, level of immunity, the virulence of the infecting parasite, and environmental factors (8). Epidemiological studies show that in areas of intense malaria transmission, immunity against severe malaria is acquired relatively rapidly (generally by the age of 5 years) (10–13) compared with immunity to uncomplicated malaria (UM), which is achieved in early adulthood (10). Immunity against asymptomatic infection is never completely achieved (10, 14). Modeling studies suggest that immunity against noncerebral forms of SM is acquired after one or two infections (11). This relatively rapid acquisition of immunity against SM lends strong support to the feasibility of developing a malaria vaccine targeting young children. Dissecting and defining the immunological basis of protection against SM is therefore vital, but for logistical reasons, it has been undertaken in only a few prospective studies (15–20).

Hospital-based studies, in which immunological responses are measured when children present with SM, are more common (21–30) and somewhat simpler to undertake. However, the ability to infer causality between an antibody measure and SM is limited, as there is no temporal relationship between the two. Not surprisingly, hospital-based studies comparing antibodies in children admitted with SM versus controls have failed to provide consistent results. While this may be attributed in part to important methodological differences between studies, a key omission has been the demonstration of the functional activity of protective antibodies.

Antigens expressed on the surface of infected red blood cells (iRBCs) are targets of antibodies that have been shown to inhibit or reverse sequestration of iRBCs, inhibit formation of rosettes (31), and promote opsonization of iRBCs for uptake by phagocytes (32, 33). Antibodies targeting the invasive merozoite stage could confer protection against SM through multiple mechanisms, including the inhibition of erythrocyte invasion and replication (34), complement-dependent mechanisms (35), and promotion of uptake and clearance by circulating leukocytes (36, 37). Although many protective mechanisms have been proposed, the majority have been studied in UM (33, 36–38) and not in SM (15). Epidemiological observations suggest that the immune mechanisms underlying the two outcomes may well be distinct, as the rates of acquisition of immunity against them differ (10). A few studies have explored antibody function in SM but have focused on the iRBC (31, 39, 40) and schizont (15) stages. The invasive merozoite stage has received less attention in this respect.

Here, we designed a case-control study of SM nested within a longitudinally monitored birth cohort of children at the Kenyan coast. We analyzed samples collected prospectively, before hospital admission, with well-characterized clinical phenotypes of SM for antibodies against five merozoite antigens, parasite schizont lysate, and the intact iRBCs. We investigated the mechanisms of action of antibodies directed against merozoites using assays of growth inhibition activity (GIA) (41) and antibody-dependent respiratory burst (ADRB) activity (42). We assessed the odds of developing SM in the first 2 years of life in the presence and absence of these immunological parameters singly and in combination.

## MATERIALS AND METHODS

### Study setting and population.

The study was conducted in Kilifi County along the coast of Kenya. Participants were drawn from within a well-established community surveillance framework known as the Kilifi Health and Demographic Surveillance System (KHDSS) (43). It covers an area of approximately 900 km^2^ around Kilifi County Hospital (KCH), which is the first-level referral facility for the county and tracks a population of approximately 260,000 individuals (43). Quarterly visits to participants' homesteads are conducted on a continuous basis to collect demographic information. The area experiences two seasonal peaks in malaria transmission (June to August and November to December) (44). A marked decline in malaria transmission has been observed in the area from the year 2002 to date (45, 46), which includes the period covered by the present analysis (2001 to 2010). The decline may be partly attributed to the scale-up of insecticide-treated bed net (ITN) usage (47) and the change of antimalarial drug policy from sulfadoxine-pyrimethamine to artemisinin-based combination therapy.

### Study cohort.

A detailed description of the study cohort has been provided elsewhere (48). Briefly, children born at KCH or attending its vaccination clinic during the first month of life were recruited into a longitudinal birth cohort (Kilifi Birth Cohort [KBC]) linked to the KHDSS to evaluate the risk factors for invasive pneumococcal disease in young children. Follow-up visits were conducted every 3 months (quarterly) for the first 2 years of life at the Outpatient Department of KCH, where axillary temperature was recorded, thick and thin blood smears for microscopy were collected, and a 2-ml venous blood sample was drawn. Children with asymptomatic parasitemia detected during the quarterly visits were not treated with antimalarial drugs. Sera and packed cells were separated and stored at −80°C. Children who did not attend the scheduled quarterly visits were visited at their homes on the subsequent day and invited to attend the next appointment.

Parents of children who were unwell outside the follow-up visits were advised to seek medical attention at KCH, and the children were treated according to the Kenya National and WHO recommended guidelines. In the event of admission to the hospital, KBC participants were identified using a unique personal identification number that links their demographic data to the clinical and laboratory information collected.

Written informed consent was obtained from the parents/guardians of all the children. Ethical approval was granted by the KEMRI National Ethics Committee.

### Study design.

We conducted a matched case-control study nested within the longitudinally monitored birth cohort described above. Index cases were children admitted between 2002 and 2010 with parasites detectable by microscopy and one of the following syndromes of severe malaria: (i) severe anemia, defined as Hb of <5 g/dl; (ii) impaired consciousness, defined as a Blantyre coma score (BCS) of less than 5; and (iii) chest indrawing or deep breathing, i.e., respiratory distress as a marker of metabolic acidosis (4). Children who fulfilled the criteria for severe malaria but, in addition, had positive cultures of blood or cerebrospinal fluid (CSF) and/or a white blood cell count of >10/μl in CSF were excluded. This was done to avoid misclassification with other potential causes of severe illness on the basis that the parasitemia may have been coincidental (49).

Cases were individually matched to a maximum of three controls by age, date of sample collection, and area of residence. Controls were selected from KBC participants who did not present to KCH with severe malaria during the 8-year period.

### Detection of P. falciparum infections.

Detection of P. falciparum in blood samples collected during the quarterly follow-up visits and during admission has been described previously (48). Briefly, thick and thin blood films were prepared, and parasite densities were determined as the number of parasites per 8,000 white blood cells per μl of blood. The prevalence of submicroscopic infections was determined by PCR (48, 50).

### Recombinant P. falciparum merozoite antigens.

We evaluated responses to eight recombinant antigens representing five merozoite targets that are currently being assessed as potential blood-stage malaria vaccine candidates. The circulating parasite population in the study area is heterogeneous based on the allelic frequencies of AMA1 (51), MSP-2 (52), and MSP-3 (53) polymorphic antigens. Consequently, we tested different allelic sequences of the antigens from various P. falciparum laboratory strains expressed in Escherichia coli or Pichia pastoris. MSP-2 antigens were based on the Dd2 and CH150/9 parasite strains and were expressed as glutathione *S*-transferase (GST) fusion proteins in E. coli (54). The 19-kDa fragment of MSP-1 (MSP-1_19_) was expressed as a GST fusion protein and was based on the Wellcome parasite strain (55). Full-length MSP-3 antigens were expressed as maltose binding protein (MBP) fusion proteins in E. coli and were based on the 3D7 and K1 parasite strains (56). AMA1 antigens were expressed in P. pastoris as His-tagged proteins and were based on the FVO, 3D7, and HB3 parasite strains (57, 58). A fragment of P. falciparum Rh2 (*Pf*Rh2) that was similar in *Pf*Rh2a and *Pf*Rh2b was based on the 3D7 parasite strain and was expressed in E. coli as a GST-fused protein (59). A P. falciparum schizont lysate based on the A4 parasite line was prepared by sonicating a highly synchronous culture containing mature schizonts (18). The lysate was stored frozen at −80°C.

### Standard ELISA.

A well-established standard enzyme-linked immunosorbent assay (ELISA) protocol was used to measure antibody titers against the recombinant antigens (60–62) and P. falciparum schizont extract (18). Wells of Dynex 4HBX Immunolon plates (Dynex Technologies Inc.) were coated with 100 μl of 0.5 μg/ml recombinant antigen diluted in coating buffer (15 mM Na_2_CO_3_, 35 mM NaHCO_3_, pH 9.4 to 9.6) or 100 μl of schizont extract diluted 1:1,000, as previously described (18). After overnight incubation at 4°C, the plates were washed four times in 1× PBS containing 0.05% Tween 20 (wash buffer) and blocked for 5 h at room temperature with 1% skim milk diluted in phosphate-buffered saline (PBS)-Tween 20 (blocking buffer). The plates were washed four times in wash buffer, and 100 μl of serum diluted 1:1,000 in blocking buffer was added to each well. Following overnight incubation at 4°C, the wells were washed and incubated for 3 h at room temperature with 100 μl of horseradish peroxidase (HRP)-conjugated polyclonal rabbit anti-human IgG (Dako) diluted 1:5,000 in blocking buffer. The wells were washed four times and incubated at room temperature with 100 μl of development buffer (0.1 M citric acid, 0.2 M Na_2_HPO_4_, 4 mg *o*-phenylenediamine dihydrochloride tablets [Sigma], 8 μl hydrogen peroxide, and 5 ml distilled water). After 15 min, the reaction was stopped with 25 μl H_2_SO_4_, and the absorbance was read at 492 nm. Eleven serial dilutions of a purified immunoglobulin reagent (malaria immune globulin [MIG]) prepared from a pool of Malawian adults presumed to be malaria immune (63) were included for every antigen tested to obtain a standard dilution curve that allowed conversion of optical density (OD) readings to antibody concentrations relative to levels present in MIG (64). For each serum, the mean OD values for the purified GST and MBP tags were subtracted from the mean OD values of the MSP-2 and MSP-1_19_ GST fusion proteins and the MSP-3 MBP fusion proteins to correct for background antibody binding to the fusion tags. Reactivity to the GST and MBP tags was found to be insignificant (OD values of <0.2). For wells where the subtraction resulted in an OD value that was less than zero, the OD value was converted to zero. A pool of sera obtained from Kilifi adults was included on a single well in each plate as a positive control to allow standardization of day-to-day and plate-to-plate variation. The coefficient of variation (CV) recorded for the serum pool across plates and across days was less than 5%. Twenty sera obtained from non-malaria-exposed United Kingdom adults were also included as negative controls for each antigen tested. For quality control, all samples were assayed in duplicate, and those that had a CV greater than 20% were repeated.

### Assay of GIA.

GIA was measured after two cycles of parasite replication according to previously established protocols (41, 65). Briefly, 50 μl of sera was dialyzed to remove antimalarial drugs prior to assay setup using 20-kDa MWCO Slide-A-Lyzer mini-dialysis tubes (Thermo Fisher Scientific). The sera were dialyzed against two changes of 1× PBS, each lasting 1 h, and a final overnight dialysis at 4°C. After dialysis, the samples were transferred to Amicon Ultra 0.5 centrifugal filter devices of 100 kDa MWCO (Millipore) and reconstituted to the original volume. Thereafter, the sera were heat inactivated for 30 min at 56°C. Forty-five microliters of highly synchronous P. falciparum 3D7 trophozoite parasites (24 to 30 h postinvasion) at 0.3 to 0.5% parasitemia and 1% hematocrit was added to individual wells of sterile 96-well U-bottom plates (Falcon). Thereafter, 5 μl of dialyzed test sera was added in duplicate, and the plates were placed in a sealed humidified gas chamber and incubated at 37°C in 5% O_2_, 5% CO_2_, and 90% N_2_. Duplicate wells with a pool of sera from nonimmune United Kingdom adults were included as a negative control and 10 mg/ml of purified MIG as a positive control. Ten microliters of fresh complete medium was added to the wells after the first growth cycle, and the plates were incubated for a further 40 h. At the end of the second growth cycle, 10 μg/ml of ethidium bromide (molecular grade) diluted in 1× PBS was added to each well, and the plates were incubated for 30 min at room temperature in the dark. The plates were centrifuged at 1,200 rpm for 1 min, and the pellet was resuspended in 200 μl of 1× PBS. Parasitemia was determined by analyzing 70,000 events on an FC500 (Beckman Coulter) flow cytometer.

### Isolation of peripheral blood PMNs.

Polymorphonuclear cells (PMNs) were isolated from whole blood collected in EDTA tubes, layered onto Polymorphprep (Axis-Shield Diagnostics) at a ratio of 1:1, and separated at 500 × *g* for 40 min at room temperature. The PMNs were collected and washed in PMN buffer [0.1% bovine serum albumin (BSA) and 1% d-(+)-Glucose Hybri Max (Sigma) in Hanks balanced salt solution (HBSS)]. Lysis of residual RBCs was performed by addition of ice-cold 0.2% sodium chloride for 30 s, followed by 1.6% sodium chloride. The cells were washed, resuspended in PMN buffer, and enumerated using a fast-read disposable chamber.

### Isolation of PEMS.

Experiments were performed with the 3D7 laboratory strain of P. falciparum. Parasitophorous-vacuole-enclosed membrane structures (PEMS) were isolated as previously described (66). Briefly, parasite cultures were routinely maintained at <10% parasitemia and 2% hematocrit. Following two rounds of synchronization by sorbitol lysis, the rings were allowed to develop into mature trophozoites and were isolated on a 65% Percoll (Sigma) density gradient. Thereafter, a cysteine protease inhibitor, *trans*-epoxysuccinyl-l-leucylamido(4-guanidino)butane (E-64; Sigma) was added for 8 h to allow growth of late-stage pigmented trophozoites into schizonts without rupture. The treated cultures were pelleted by low-speed centrifugation, resuspended in PBS at 1.8 × 10^6^ schizonts/ml, and immediately frozen at −20°C to lyse the RBCs.

### Assay of ADRB activity.

To assess ADRB activity, frozen PEMS aliquots were thawed, and individual wells of Nunc opaque MaxiSorp 96-well plates were coated at 1.8 × 10^5^ schizonts/ml. The wells were washed three times with 1× PBS and blocked for 1 h at 37°C with casein (Thermo Scientific). One hundred microliters of test serum diluted 1:50 in 1× PBS was added to individual wells in duplicate and incubated for 1 h at 37°C. Three washes were performed, followed by addition of 50 μl of 4-aminophthalhydrazide (Sigma) at 0.04 mg/ml and 50 μl PMNs at 1 × 10^7^ cells/ml. Each serum sample was assayed in duplicate using PMNs from a single donor. The plates were immediately read on a Varioskan flash luminometer (Thermo Scientific). Chemiluminescence readings from each well were obtained for 1 s every minute for an hour (42, 67). The results are presented as relative light units (RLU) indexed to the readings of MIG (42, 67).

Duplicate wells containing a pool of malaria-semi-immune and -naive sera were included for each individual neutrophil donor to assess donor variability. The indexed RLU values of the malaria-naive and -semi-immune sera were 0.065, 0.093, 0.100, 0.104, 0.071, 0.107, and 0.063 and 0.997, 0.793, 0.718. 0.750, 0.851, 0.799, and 0.865, respectively, when tested against seven independent neutrophil donors. Although the absolute indexed RLU values appeared to vary between donors, the ranking of the ADRB activities of these controls was consistent across different days of assay setup, and we could distinguish between responders and nonresponders (based on the cutoff of the mean plus 3 standard deviations of malaria-naive sera).

### Flow cytometry.

Levels of IgG (measured as median fluorescence intensity [MFI]) against the surface of intact RBCs infected by the 3D7 laboratory strain were determined using flow cytometry, as previously described (68). Briefly, mature trophozoites were obtained at 1% to 5% parasitemia and resuspended at 1% hematocrit in 0.1% BSA-PBS. Thereafter, 1 μl of plasma and 9 μl of parasite suspension were added to each well of a 96-well flat-bottom plate (Nunc) and incubated at room temperature for 30 min. The wells were washed three times with 0.1% BSA-PBS, and the contents were resuspended in 25 μl 0.1% BSA-PBS containing rabbit anti-human IgG at a concentration of 1:100 and incubated for 1 h at room temperature. After three washes, 25 μl of 0.1% BSA-PBS containing fluorescein isothiocyanate (FITC)-conjugated goat anti-rabbit IgG at 1:50 dilution and 10 μg/ml of ethidium bromide were added to each well. The plates were incubated for 1 h at room temperature in the dark, after which three washes were performed. Based on data from previous studies, the acquisition of 1,000 trophozoite-infected erythrocytes enabled a clear distinction between positive and negative populations after incubation with malaria-immune sera (68). Here, at least 100,000 events representing 1,000 iRBCs were acquired on an FC500 flow cytometer (Beckman Coulter) and analyzed using FlowJo software. The MFI for each sample was obtained by subtracting the MFI of uninfected erythrocytes from that of infected erythrocytes. Ten sera obtained from non-malaria-exposed United Kingdom adults were also included as negative controls. The mean background MFI based on the malaria-naive sera was 4.2 (range, 2.12 to 6.23).

### Statistical analysis.

Data analysis was performed using STATA 11.2 (StataCorp, College Station, TX). Antibody seropositivity was defined as a cutoff above the mean plus 3 standard deviations of 20 non-malaria-exposed United Kingdom adult sera. An individual was classified as a high or low GIA responder if their GIA was above or below the median GIA level, respectively. Seropositivity for ADRB was defined as a cutoff above the mean plus 3 standard deviations of 10 healthy malaria-naive United Kingdom sera obtained from phase I/IIa malaria vaccine trials at the Jenner Institute, University of Oxford (69, 70). Seropositivity to the intact iRBCs was defined as MFI levels above the mean plus 3 standard deviations of 10 malaria-naive United Kingdom adult sera. Antibody levels were compared using the Mann-Whitney or Kruskal-Wallis test. The correlations between antibody levels, GIA levels, and ADRB levels were determined using Spearman's rank correlation.

We have previously defined threshold antibody concentrations that are associated with protection against uncomplicated malaria episodes in two independent cohorts (64, 71). Here, we tested whether children who attained antibody levels above the proposed thresholds also had reduced odds of developing SM.

Conditional logistic regression (CLR) models were used to derive odds ratios (OR) and 95% confidence intervals (CI) using data from risk sets that had at least one matched control per case (48). A risk set was defined as an index case and his or her individually matched controls. We observed marked differences in parasite prevalence between the cases and controls, despite our efforts to minimize differences in parasite exposure by closely matching the index cases to their corresponding controls by age and residence. To account for the observed residual confounding, antibody titers to schizont extract (as a continuous variable) were included in the CLR model.

## RESULTS

### Baseline characteristics of SM cases and their corresponding controls.

**FIG 1:**
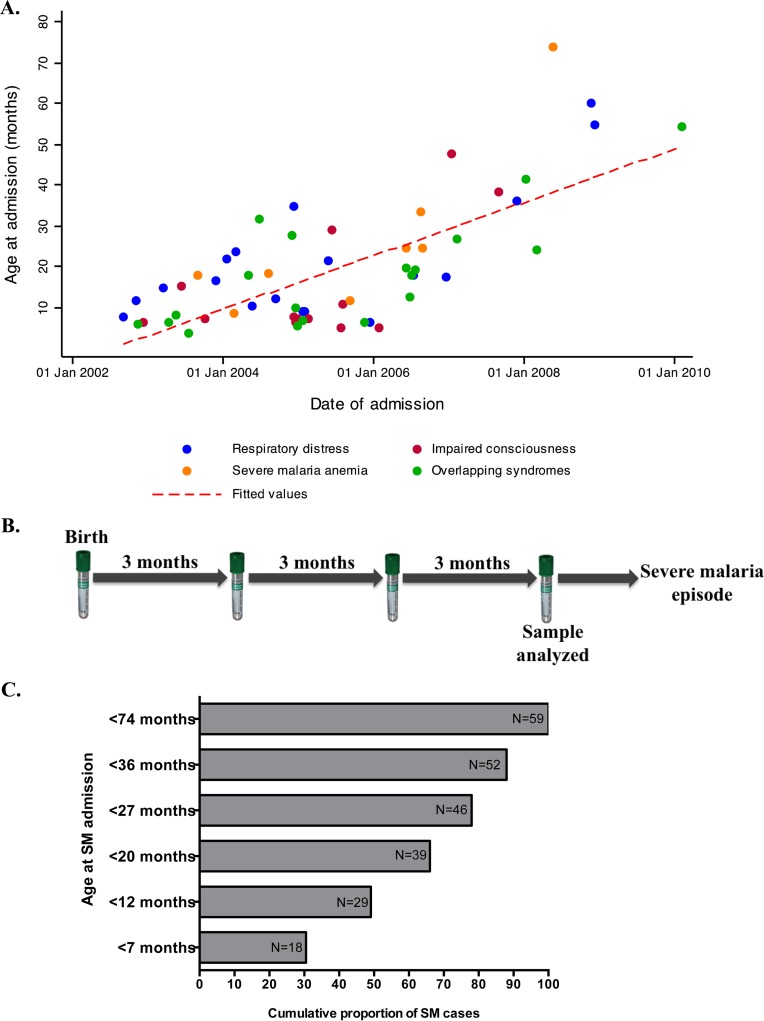
Characteristics of severe malaria cases and sampling criteria. (A) Ages at admission with different SM syndromes during the 8-year follow-up period of the study (2002 to 2010). The red dashed line shows the linear regression fit of age at admission with different SM clinical phenotypes on the year of follow-up. (B) Illustration of the sampling criteria showing the quarterly visits at which a blood sample was collected and the specific time point selected for antibody measurements. (C) Cumulative proportions of SM cases recorded at different ages.

A detailed profile of the birth cohort illustrating the selection criteria for severe malaria patients and their corresponding controls has been published (48). A total of 61 SM cases were identified, 19 (31.1%), 15 (24.5%), and 8 (13.1%) of whom presented at the hospital with respiratory distress (deep breathing or chest indrawing), impaired consciousness (BCS < 5), and severe malaria anemia (SMA) (Hb < 5g/dl), respectively. Fourteen (22.9%) presented with two overlapping SM syndromes and 5 (8.1%) with all three syndromes (48). The median age at admission was 19.3 (range, 5.0 to 74.0) months. Four children died at the time of admission with SM. We observed a gradual shift in the age of SM admission, from younger to older children, over time between 2002 and 2010 (*r_s_* = 0.52; *P* < 0.0001) (Fig. 1A). During this period, a significant decline in malaria transmission in Kilifi County was reported (45). Interestingly, the proportion of children admitted to the hospital with different SM syndromes was independent of age at the time of admission (Fig. 1A).

Samples were collected every 3 months from birth up to 2 years of age. The analysis presented here is drawn from antibody responses measured in serum samples collected at the time point immediately prior to the severe malaria episode for cases and their individually matched controls, as illustrated in Fig. 1B.

The median time between date of sampling and date of index case admission was 2.7 (range, 0.1 to 21.5) months and 2.5 (range, 0.1 to 21.2) months for cases and corresponding controls, respectively (Table 1). Of the 61 SM cases identified, serum samples collected at the sampling time point prior to the SM episode were available for 59 children. They were matched on age, date of sample collection, and location of residence to a maximum of three controls. A total of 161 controls were identified, from whom 147 serum samples were available for analysis.

**TABLE 1:** Baseline characteristics of the study population

Characteristic	Value	
	Cases (*n* = 59)	Controls (*n* = 161)
Female/male no. (ratio)	29/30 (0.9)	70/78 (0.8)
Age (mo) at index SM case admission^*a*^ [median (range)]		
All SM admissions (*n* = 59)	19.3 (5.0–74.1)	20.4 (3.4–74.9)
SM cases admitted within 2 yr and 3 mo of age (*n* = 46)	10.1 (5.8–24.1)	9.3 (6.3–19.8)
Duration (mo) between date of sample collection and index SM case admission^*a*^ [median (range)]	2.7 (0.1–21.5)	2.5 (0.1–21.2)
Proportion parasite positive^*b*^ during any visit prior to case admission [*n*/total (%)]	21/170 (12.3)	36/458 (7.8)
Proportion parasite positive at the visit immediately preceding case admission [*n*/total (%)]	9/58 (15.5)	6/144 (4.1)
Proportion of individuals with fever^*c*^ and any parasitemia during any visit [*n*/total (%)]	5/165 (3.0)	12/427 (2.8)

a Severe malaria cases admitted within 2 years and 3 months of age (*n* = 46).

b Parasite positive by either microscopy or PCR.

c Fever was defined as a temperature of >37.5°C.

The female/male ratio, median age at the time of index case admission, and proportion of children who had fever and asymptomatic parasitemia during the quarterly visits were similar between the cases and controls (48) (Table 1). A total of 46 (75.4%) cases were admitted with severe malaria within 27 months of age (Fig. 1C). The remaining 13 cases aged between 27.7 and 74.0 months were excluded from the present analysis because it was considered highly unlikely that an antibody response measured from samples collected during the first 2 years of life would predict the risk of disease up to 6 years later. Antibodies against merozoite antigens are relatively short-lived in children (72). The proportion of children who were parasite positive at the visit preceding index case admission was higher in the cases (15.5%) than in the controls (4.1%). However, the difference was not statistically significant. A similar pattern was also observed when all the visit samples collected from birth up to the time of index SM case admission were considered (48) (Table 1).

We also compared the dynamics of the antibody titers between the cases and controls using samples collected at all the 3-month time points before the admission of the cases (see Fig. S1 in the supplemental material). A large proportion of the children had low antibody titers, often fluctuating below the seropositivity cutoff at all the time points. Some children had detectable antibody levels that often fluctuated either above the cutoff (i.e., they were always seropositive, but the actual levels varied) or around the cutoff (i.e., they were occasionally seropositive or seronegative at the different time points). Importantly, the individual patterns of antibody responses were similar between the cases and controls for the antigens tested (see Fig. S1 in the supplemental material).

### Age-specific antibody titers to P. falciparum merozoite antigens, iRBCs, GIA, and ADRB among cases and controls.

**FIG 2:**
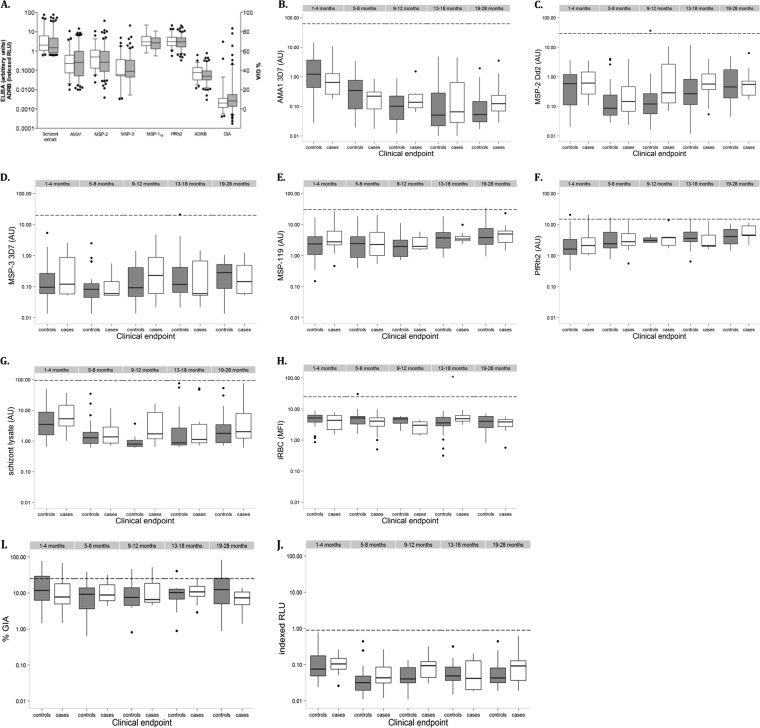
Preexisting antibodies against P. falciparum merozoite antigens, GIA, and ADRB activity among the cases and controls. (A) Overall antigen-specific IgG levels, GIA, and ADRB activity between the controls (gray boxes) and cases (white boxes). (B to H) Age-specific antibody levels against AMA1 3D7 (B), MSP-2 Dd2 (C), MSP-3 3D7 (D), MSP-1_19_ (E), *Pf*Rh2 (F), parasite schizont lysate (G), and intact iRBC surface antigens (H). (I and J) Age-specific GIA (I) and ADRB activity (J). The horizontal dashed lines represent the levels from a pool of semi-immune adults resident in Kilifi. Data are presented as box-and-whisker plots (boxes show medians and interquartile ranges; whiskers show the maximum and minimum values). Filled circles represent outlier values.

Data are presented for measurements obtained at the quarterly sampling point prior to development of severe malaria for index cases and their individually matched controls. Because antibody reactivities to allelic forms of AMA1 (*r_s_* = 0.94 for FVO versus 3D7, *r_s_* = 0.85 for HB3 versus FVO, and *r_s_* = 0.82 for HB3 versus 3D7; *P* < 0.001), MSP-2, and MSP-3 were strongly correlated (*r_s_* = 0.67 and 0.53, respectively; *P* < 0.001), responses to only one allelic form of these antigens are shown in Fig. 2. In general, antibody titers to all antigens were markedly lower in children aged 0 to 2 years than in a pool of sera obtained from adults resident in the same area (Fig. 2). We also determined the number of children who had antibodies above the thresholds proposed to be important for protection against clinical malaria (64, 71) and found that only two children had antibodies above the threshold (one against MSP-2 and the other against MSP-3). These two children did not develop severe malaria during the 2-year follow-up period. There was no significant difference in the levels of GIA, ADRB, and antibody titers to schizont extract, AMA1, MSP-2, MSP-3, MSP-1_19_, and *Pf*Rh2 between the index cases and controls (Fig. 2A).

Overall antibody levels were highest in children below 6 months of age, with the exception of anti-MSP-1_19_ and anti-*Pf*Rh2 antibodies (Fig. 2). Thereafter, the levels declined with increasing age to a plateau at 13 to 18 months for AMA1 (Fig. 2B) and 5 to 8 months for MSP-2 (Fig. 2C), MSP-3 (Fig. 2D), and parasite schizont lysate (Fig. 2G). This was followed by a steady increase in levels with age in both cases and controls. Age-specific comparisons of antibody levels against *Pf*Rh2 and MSP-1_19_ between the cases and controls revealed a steady rise in antibody levels with increasing age from birth until 2 years of age (Fig. 2E and F). There was no significant difference in IgG binding to the intact iRBC surface between the cases and controls compared within the different age groups (Fig. 2H).

Interestingly, in contrast to all other antibody measurements, the level of GIA in young children under the age of 2 was similar to that observed in semi-immune adults (Fig. 2I). Both GIA and ADRB levels were highest in the 1- to 4-month-old children and declined to a plateau at 5 to 8 months, followed by a steady increase in both cases and controls (Fig. 2I and J).

We also compared antibody levels between children who had concurrent parasitemia (either by microscopy or PCR) and those who were parasite negative (see Fig. S2 in the supplemental material). IgG levels were higher in the children who were parasite positive than in those who were parasite negative across the age categories for all the antigens tested. However, this difference was significant only in children over 13 months of age for anti-AMA1, anti-MSP-2, anti-MSP-3 and anti-parasite schizont lysate antibodies (see Fig. S2A, B, C, and F in the supplemental material). In contrast, GIA levels were consistently higher in the parasite-negative children than in the parasite-positive children (see Fig. S2H in the supplemental material), and an opposite trend was observed for ADRB activity (see Fig. S2I in the supplemental material). However, neither of these differences was statistically significant.

### Relationship between different indices of immunity in children aged 0 to 2 years.

We investigated the correlations between the different indices of the antimalarial immune response that we had assessed in this study. There was a significant positive correlation between IgG responses to schizont extract and to AMA1 and MSP-2 (*r_s_* > 0.50; *P* < 0.05) for both cases (Table 2) and controls (Table 3), suggesting that of our panel of antigens, they are among the most recognized in the schizont extract preparation. To determine the pattern of responses across antigens for each individual, the range of antibody concentrations was divided into quartiles and displayed on a plot matrix (see Fig. S3 in the supplemental material). AMA1 responses were strongly correlated with MSP-2 and MSP-3 responses in both the cases (Table 2) and controls (Table 3), with approximately 50% and 30% of strong responders to AMA1 alleles also responding strongly to MSP-2 and MSP-3 alleles, respectively (see Fig. S3 in the supplemental material). These findings suggest that in young children, antibodies to AMA1, MSP-2, and MSP-3 are coacquired.

**TABLE 2:** Correlation between different indices of immunity among cases

Index	Correlation coefficient^*a*^								
	Schizont extract	AMA1	MSP-2	MSP-3	MSP-1_19_	*Pf*Rh2	iRBC	GIA	ADRB
Schizont extract									
AMA1	**0.63**								
MSP-2	**0.61**	**0.59**							
MSP-3	**0.36**	**0.49**	**0.47**						
MSP-1_19_	**0.28**	0.17	**0.31**	0.12					
*Pf*Rh2	0.04	−0.01	0.20	**0.36**	**0.31**				
iRBC	0.14	−0.05	−0.05	**−0.29**	0.08	−0.14			
GIA	−0.06	−0.17	−0.13	**−0.39**	0.13	−0.17	**0.31**		
ADRB	**0.67**	**0.70**	**0.62**	**0.60**	0.18	0.21	−0.15	−0.24	

a Correlation coefficients were calculated using Spearman's rank correlation test. Statistically significant values at a *P* value of <0.05 are shown in boldface.

**TABLE 3:** Correlation between different indices of immunity among controls

Index	Correlation coefficient^*a*^								
	Schizont extract	AMA1	MSP-2	MSP-3	MSP-1_19_	*Pf*Rh2	iRBC	GIA	ADRB
Schizont extract									
AMA1	**0.62**								
MSP-2	**0.52**	**0.34**							
MSP-3	**0.23**	**0.23**	**0.27**						
MSP-1_19_	**0.24**	0.02	**0.34**	−0.03					
*Pf*Rh2	0.08	−0.14	**0.22**	0.13	**0.44**				
iRBC	−0.14	−0.11	−0.16	−**0.37**	0.006	−0.08			
GIA	**0.23**	**0.31**	**0.33**	0.04	**0.29**	0.01	−0.004		
ADRB	**0.57**	**0.49**	**0.44**	**0.33**	**0.20**	0.04	**−0.21**	**0.29**	

a Correlation coefficients were calculated using Spearman's rank correlation test. Statistically significant values at a *P* value of <0.05 are shown in boldface.

There was a negative correlation between antibodies to the iRBC surface antigens and the merozoite antigens tested in both the cases and controls (Tables 2 and 3). We observed no positive correlation between GIA and antibodies to any of the antigens tested among the index cases (Table 2). Among the control subjects, the correlation was weak but significantly positive, with the exception of responses to MSP-3 and *Pf*Rh2 (Table 3). In contrast, we observed a strong and significant positive association between the levels of ADRB and antibodies to all the antigens tested among both the cases (Table 2) and controls (Table 3), with the exception of both MSP-1_19_ and *Pf*Rh2 for the cases and *Pf*Rh2 for controls. Finally, we determined whether the antibodies tested in the two assays of antibody function were correlated. We found no association between the two assays among the case subjects (*r_s_* = −0.24; *P* = 0.35) (Table 2) and the controls (*r_s_* = 0.29; *P* = 0.002) (Table 3).

### Relationship of the breadths of anti-merozoite antibodies with GIA and ADRB.

**FIG 3:**
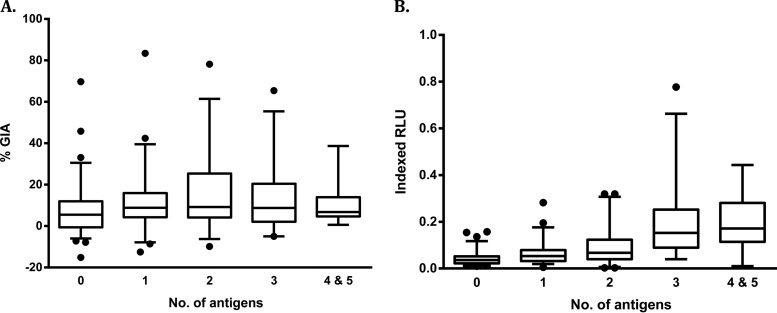
Relationship between antibody function and breadth of anti-merozoite antibody responses. Shown are the levels of GIA (A) and ADRB activity (B) according to the number of P. falciparum merozoite antigens recognized (responses above the mean plus 3 standard deviations of 20 European sera). Data are presented as box-and-whisker plots (boxes show medians and interquartile ranges; whiskers show the maximum and minimum values). Filled circles represent outlier values.

Our previous study demonstrated that the breadth of anti-merozoite antibody responses was associated with better antibody function (as measured by opsonic phagocytosis of merozoites) (36). Here, we examined whether antibody breadth (defined as the sum of responses to the five merozoite antigens) was also associated with better function as measured by GIA and ADRB activity. There was no significant rise in GIA levels with increase in antibody breadth (Kruskal-Wallis test; *P* = 0.14) (Fig. 3A). In contrast, the levels of ADRB activity rose steadily with increasing breadth of antibody responses, and this trend was statistically significant (Kruskal-Wallis test; *P* < 0.001) (Fig. 3B).

### Association between antibody titers, GIA, ADRB activity, and protection against severe malaria.

We examined the association between antibody titers (measured at the time point closest to admission) to different merozoite antigens, IgG to the intact iRBCs, GIA, and ADRB activity with the odds of developing SM during the first 2 years of life. Antibody seropositivity to all the antigens tested was not associated with protection against developing SM, with the exception of responses to AMA1 (FVO and 3D7), which were associated with a significant reduction in the odds of developing SM (OR = 0.40, 95% CI = 0.16 to 0.98, *P* = 0.04 and OR = 0.40, 95% CI = 0.17 to 0.98, *P* = 0.04, respectively) in multivariate analysis (Table 4). After adjustment for antibody responses to all the other merozoite antigens, AMA1 antibodies remained significantly associated with protection (OR = 0.37; 95% CI = 0.15 to 0.90; *P* = 0.029). Antibodies to the iRBCs were not associated with protection (OR = 0.81; 95% CI = 0.33 to 2.01; *P* = 0.48).

**TABLE 4:** Associations between different indices of immunity and odds of developing SM

Index	Univariate analysis^*a*^		Multivariate analysis^*b*^	
	OR (95% CI)	*P* value	OR (95% CI)	*P* value
AMA1				
FVO	0.53 (0.23–1.18)	0.12	**0.40 (0.16–0.98)**	**0.04**
3D7	0.51 (0.22–1.17)	0.11	**0.40 (0.17–0.98)**	**0.04**
HB3	0.55 (0.24–1.24)	0.15	0.43 (0.17–1.04)	0.06
MSP-2				
Dd2	1.22 (0.54–2.78)	0.62	1.12 (0.48–2.62)	0.78
CH150/9	1.05 (0.42–2.60)	0.90	0.92 (0.36–2.36)	0.87
MSP-3				
3D7	1.61 (0.64–4.01)	0.30	1.44 (0.56–3.70)	0.44
K1	1.33 (0.47–3.76)	0.58	1.16 (0.39–3.40)	0.77
MSP-1_19_	1.56 (0.20–11.69)	0.66	1.50 (0.20–11.00)	0.68
*Pf*Rh2	1.08 (0.40–2.90)	0.86	1.12 (0.41–3.05)	0.81
iRBC	0.73 (0.30–1.77)	0.48	0.81 (0.33–2.01)	0.65
GIA	0.62 (0.29–1.32)	0.21	0.55 (0.26–1.20)	0.13
ADRB	1.00 (0.34–2.86)	1.00	0.51 (0.14–1.88)	0.31
GIA or ADRB	0.87 (0.43–1.76)	0.71	0.68 (0.32–1.44)	0.32
GIA and ADRB	0.26 (0.03–2.22)	0.31	**0.07 (0.006–0.82)**	**0.03**

a A conditional logistic regression model was used to calculate the prospective odds of developing severe malaria in young children during the first 2 years of life.

b Reactivity to P. falciparum schizont extract (fitted as a continuous covariate) was included in the model to account for differences in parasite exposure between the cases and controls. Boldface data represent statistically significant associations (*P* < 0.05).

To determine whether the protective effects of antibodies were comparable across individual syndromes of severe malaria, separate analyses were conducted for the outcome of impaired consciousness (*n* = 31) versus noncerebral malaria (*n* = 28) (see Table S1 in the supplemental material) and SMA (*n* = 18) versus non-SMA (*n* = 41) (see Table S2 in the supplemental material). There was no association between antibodies to all the antigens tested with regard to protection against either impaired consciousness (see Table S1 in the supplemental material) or SMA (see Table S2 in the supplemental material).

Next, we examined whether the breadth of antibody responses was associated with reduced odds of developing SM. Our analysis revealed that increasing breadth of responses was not associated with reduced odds of developing SM during the first 2 years of life (see Fig. S4 in the supplemental material). In contrast, antibodies to one antigen were significantly associated with protection (OR = 0.36; 95% CI = 0.13 to 0.98; *P* = 0.04), which possibly reflects the protection accorded by anti-AMA1 antibodies in this study (Table 4).

Both GIA and ADRB activity were independently not associated with reduced odds of developing severe malaria (OR = 0.55, 95% CI = 0.26 to 1.20 and OR = 0.51, 95% CI = 0.14 to 1.88, respectively). Remarkably, children classified as being positive responders to a combination of both functional assays had very low and significantly reduced odds of developing severe malaria (OR = 0.07; 95% CI = 0.006 to 0.82) during the follow-up period compared to children who had responses to either of the two functional assays (OR = 0.68; 95% CI = 0.32 to 1.44) (Table 4).

### Role of maternal antibodies in mediating protection.

Following from our observation that the highest antibody levels were observed in children less than 6 months of age, we tested whether our significant results described above were driven by the presence of maternally transferred antibodies. First, we investigated the proportion of children who were seropositive for AMA1 and were below 6 months of age. Among those who did not develop SM, 91.8% were seropositive for AMA1 and were below 6 months of age compared to 50.0% among the index cases. The difference in proportions was statistically significant (*P* < 0.001).

Second, we tested whether the protective effect of achieving positive responses in both GIA and ADRB was driven by the presence of maternal antibodies. We compared the proportions of cases and controls who were positive responders in both assays and were less than 6 months of age. Sixty-six percent of the children who did not subsequently develop SM and were below 6 months of age were classified as positive responders in both functional assays compared to 50.0% among the cases. This difference was not statistically significant (*P* = 0.33).

## DISCUSSION

Although the vast majority of deaths due to SM occur in young children, few studies have examined the host immunological factors that might contribute to susceptibility to severe illness or resistance in the age group (15–19). Two main findings emerge from our analyses. First, we provide the first epidemiological evidence that a combination of antibodies that mediate both GIA and ADRB activity are associated with a strong and significant reduction in the odds of developing SM during the first 2 years of life. These data suggest that studying individual mechanisms of antibody function in isolation may be insufficient and potentially misleading with regard to understanding naturally acquired immunity against malaria. Second, we show that antibodies to AMA1 significantly reduced the odds of developing SM in children in this age category, suggesting it may still be viable as a vaccine candidate for very young children. Importantly, levels of anti-AMA1 antibodies and those mediating the combined functional mechanisms were highest in infants less than 6 months of age, supporting the view that passively transferred maternal IgG contributes to protective immunity. Of particular interest is the fact that our study was conducted during a period of epidemiological transition, with a decline in the intensity of malaria transmission (45). As expected, and as has been argued, we found a concurrent increase in the age of presentation at the hospital with SM (1, 45, 73). These results have broad implications for other settings in sub-Saharan Africa experiencing a similar epidemiological transition (74) and are particularly pertinent as many countries enter the preelimination phase of malaria control (75).

The GIA assay is currently the most established and widely used *in vitro* assay for measuring the functional activity of naturally acquired and vaccine-induced antibodies (76). However, prospective studies in children and adults have provided inconsistent evidence of an association between GIA and the risk of acquiring asymptomatic infection (77) or developing uncomplicated malaria (37, 65, 78–81). While there are considerable methodological differences that may account for the disparities between studies, it is still unclear whether GIA is a major contributor to naturally induced immunity to malaria (76). Other mechanisms of protection may also be important, but only a few have been analyzed in a limited number of studies (36–38). Our results suggest at least two distinct mechanisms via which antibodies may exert protective effects against the most severe outcome of infection with P. falciparum. These mechanisms may potentially act to limit the exponential growth of parasites, which is often linked to the development of SM (12).

Levels of antibodies to all the antigens tested here were low compared to those in semi-immune adults resident in the same area and even lower than the threshold concentrations we have previously reported to be important for protection against clinical malaria in this area (64). A careful review of the literature revealed that this was to be expected. Even in areas with malaria transmission intensity higher than that in our study, antibody levels have been found to be low in children under the age of 2 years (82, 83). Thus, the combination of young host age and declining malaria transmission intensity probably accounts for the low levels of antibodies we observed.

Despite these low antibody levels, we show for the first time that anti-AMA1 antibodies were independently associated with significant protection against SM in this age group. This result remained significant and strong even after adjustment for responses to all the other merozoite antigens analyzed here, as well as prior exposure defined by schizont ELISA reactivity. Our findings lend further support to a renewed interest in improving AMA1-based vaccines by demonstrating that such a vaccine could potentially protect against the most severe forms of malaria in the youngest children at the highest risk of death. AMA1 antibodies may protect by inhibiting the essential process of erythrocyte invasion (58). However, we have shown a lack of correlation between anti-AMA1 antibodies and GIA in this study, and some field studies have reported a paradoxical decrease in GIA activity with age (77, 81, 84). These findings may be explained by the presence of other naturally acquired malaria-specific antibodies, which interfere with the invasion-blocking activity of anti-AMA1 antibodies (85). It is not clear how this interference is mediated (86) or whether these findings are generalizable in all settings. The observation that anti-AMA1 antibodies are strongly correlated with ADRB activity suggests that they may contribute to antiparasitic activity. Extensive parasite polymorphism at this locus, even in parasites from children with SM (51), is a challenge for AMA1-based vaccines (87, 88). However, recent studies suggest that this seemingly insurmountable hurdle might be overcome by including a limited number of AMA1 alleles in a multiallelic vaccine (89, 90). Rhoptry neck protein 2 (RON2), another parasite protein released from the rhoptries prior to invasion, has been shown to interact with the conserved hydrophobic pocket of AMA1, triggering tight-junction formation, an irreversible step that commits the parasite to invasion. Disrupting this interaction with a RON2 peptide (RON2L) is sufficient to disrupt parasite invasion (91). Furthermore, immunization with an AMA1-RON2 peptide complex provided complete antibody-mediated protection against a lethal Plasmodium yoelii challenge (92). In their study, Srinivasan et al. demonstrated levels of growth-inhibitory antibodies in mice vaccinated with the complex that showed enhanced invasion inhibition compared to those in mice vaccinated with AMA1 alone, and this was related to a switch in the proportion of antibodies binding to less polymorphic loops surrounding the hydrophobic pocket, which binds RON2 (92). These findings suggest that the efficacy of multiallelic AMA1 subunit vaccines may be further improved if they are formulated in complex with RON2L. Preexisting antibodies to MSP-2, MSP-3, MSP-1_19_, and *Pf*Rh2 were not independently associated with reduced odds of developing SM, also reported independently for antibodies against MSP-1_19_ and MSP-3 (15).

We have previously argued that high levels of antibodies against merozoite antigens are required for protection against clinical episodes of both UM and a less rigorously defined SM (62, 64). The current study challenges that view, demonstrating that although levels appeared to be low, functional antibodies could still be detected that in combination were remarkably strongly associated with protection. As recently reported for opsonic phagocytosis assays (36), the breadth of the anti-merozoite antibody response correlated with the amount of reactive oxygen species production in the ADRB assay. This lends support to the idea that cumulative responses act synergistically to mediate protection (93, 94) and is the first demonstration of this principle with regard to antibody function and in SM. It of course remains possible that young children develop high-titer responses to other antigens not analyzed here, and one priority is to study a wider range of merozoite targets in relation to SM. Of note, we did not find any evidence that antibodies against the surface of intact iRBCs were associated with protection, contrary to what has been previously demonstrated for SM (16) and UM (33). However, we tested IgG binding to the iRBC surface of only one laboratory strain (3D7), which may inadequately represent the diversity of parasite proteins expressed on infecting parasite populations.

Notably, GIA and ADRB were poorly correlated, as has been reported for the relationship between GIA and opsonic phagocytosis of merozoites (36). Our data suggest that the targets of GIA and ADRB are likely to be different but that the net effect of both is complementary and raise the possibility that studying even more mechanisms may be highly informative for understanding immunity. Similarly, the correlations between the specific merozoite responses analyzed here and functional assays were even weaker than those recently reported for opsonic phagocytosis of merozoites (36). This could be partly explained by the low levels of antibodies in the present study but suggests that the “true” targets of protective immunity against SM have yet to be identified and that these functional assays can be used for this purpose.

Maternal antibodies are widely believed to play a key role in protection among infants in multiple infectious diseases (95–97); however, direct evidence in support of this for malaria has been limited (98). Previous studies have yielded discrepant results (reviewed in reference 98), possibly due to methodological differences, including the diversity of malaria antigens tested, variations in malaria transmission intensity, inconsistent periods of observation, and disparities in the primary outcomes assessed. A major omission from all studies to date is the failure to examine the contributions of functional mechanisms that may predict protection. Though our numbers were limited, we observed that a higher proportion of infants less than 6 months of age who did not develop SM had functionally active antibodies than those who developed SM. This result was not statistically significant but suggests that the functional quality of passively transferred maternal antibodies may be important.

SM often presents in three major overlapping clinical syndromes in areas with high and sustained levels of malaria transmission intensity: impaired consciousness, respiratory distress, and severe malaria anemia (4). The presentation of these clinical features varies; in areas of high transmission intensity, younger children present primarily with severe anemia, while in low-transmission-intensity regions, cerebral malaria in older children is predominant (2, 3). Despite a shift in the age of hospital admissions with SM to older children in our study, cerebral malaria was not the dominant disease manifestation, contrary to expectations (45). We have previously reported more genetically diverse infections in the group of children who were admitted with severe noncerebral malaria, suggesting differences in immune control of infections (48). However, we found no evidence that the targets and mechanisms that protect against CM versus SMA were different, although our study was not adequately powered to investigate the individual syndromes within SM. Further studies are needed to address this gap in knowledge and to determine whether they vary in different transmission settings.

Finally, it has been argued that a decline in malaria transmission intensity, such as was observed in our setting and has been reported from many parts of Africa, could have detrimental effects on naturally acquired immunity (1). This is partly borne out in our study, where we observed a shift in the burden of SM from younger to older children, in keeping with what has previously been reported in this area with regard to all malaria admissions (45, 73). There have also been concerns that interventions that reduce malaria transmission may lead to a paradoxical upsurge in mortality associated with severe complications of cerebral malaria (99). We did not observe this in our study. The case fatality rate was low, with only four SM-related deaths over an 8-year follow-up period. However, these findings should be interpreted with caution, since parents/guardians were encouraged to bring their children to the hospital as soon as they suspected the child was unwell, which may have influenced the natural course of the disease. The children were also actively monitored every 3 months for any suspected illness, reducing the chances of fatal outcomes. Data on the use of antimalarial drugs outside the hospital setting were also not available. It also remains possible that deaths due to SM could have occurred after completion of the 2-year follow-up period and were therefore not captured.

In summary, we provide the first evidence that antibodies against AMA1 and the combined ability of total IgG to inhibit parasite growth and to mediate the release of reactive oxygen species from PMNs were remarkably strongly associated with reduced odds of developing SM in African children less than 2 years of age. Assessment of a combination of assays that measure multiple mechanisms of antibody function could be useful *in vitro* correlates of immunity to objectively prioritize known and predicted antigens being evaluated as vaccine targets in preclinical and clinical studies.

## Supplementary Material

Supplemental material
