# Supplementary material for: Targets and Mechanisms Associated with Protection from Severe Plasmodium falciparum Malaria in Kenyan Children
Source: Infect Immun. 2016 Mar 24;84(4):950–63. doi: 10.1128/IAI.01120-15 (PMC4807498; doi:10.1128/IAI.01120-15)
Supplement: Supplemental material [file supp_84_4_950__index.html]

Supplemental material 

# Severe *Plasmodium falciparum* malaria: targets and mechanisms associated with protection in Kenyan children

## Supplemental material

- Supplemental file 1 -

  Fig. S1. Dynamics of individual antibody titers between the SM cases and controls. Fig. S2. Age-specific antibody titers, GIA levels, and ADRB activity. Fig. S3. Plot matrix showing each individual’s response against schizont extract, AMA1 (3D7), MSP-2 (Dd2), MSP-3 (3D7), MSP-119, PfRh2, and binding to the iRBC surface. Fig. S4. Protective efficacy of antimerozoite antibodies according to the number of antigens recognized. Table S1. Association between antibody levels to specific merozoite antigens, the intact iRBC, GIA and ADRB activity, and the odds of developing impaired consciousness. Table S2. Association between antibody levels to specific merozoite antigens, the intact iRBC, GIA and ADRB activity, and the odds of developing SM anemia.

  PDF, 1.7M
