# Supplementary material for: Targets and Mechanisms Associated with Protection from Severe Plasmodium falciparum Malaria in Kenyan Children
Source: Infect Immun. 2016 Mar 24;84(4):950–63. doi: 10.1128/IAI.01120-15 (PMC4807498; doi:10.1128/IAI.01120-15)
Supplement: Supplemental material [file IAI.01120-15_zii999091636so1.pdf]

Supplementary Figure 1

Dynamics of individual antibody titres between the SM cases and controls. Each line represents the pattern of antibody titres for an individual over time against, **(A-B)** AMA1 (3D7), **(C-D)** MSP-2 (Dd2), **(E-F)** MSP-3 (3D7), **(G-H)** MSP-1_{19}, and **(I-J)** *Pf*Rh2 plotted separately for the cases and controls. Horizontal red lines represent the seropositivity cutoff defined as mean+3SD of twenty European plasma.

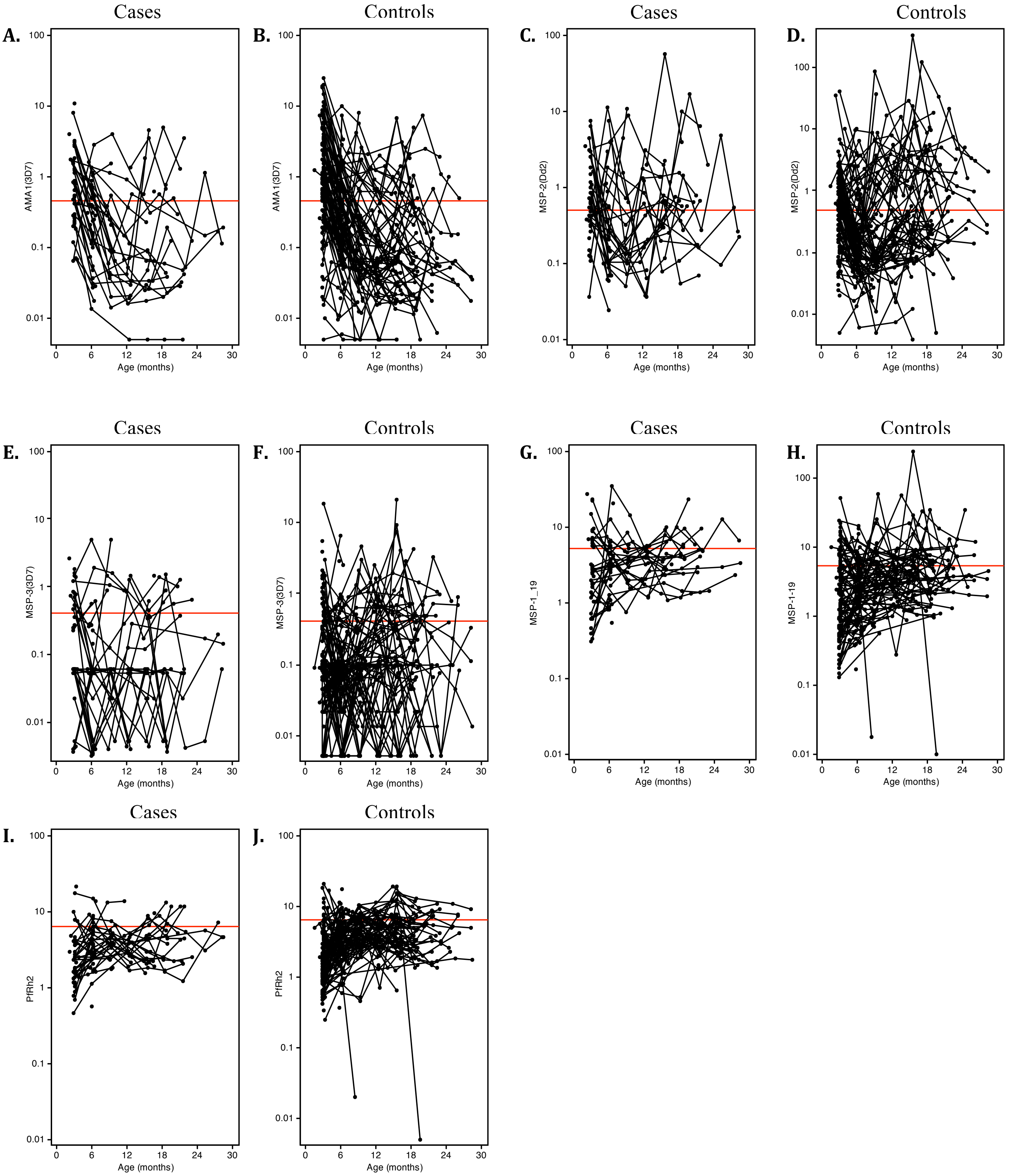

A.
Cases
AMA1(3D7)
100
10
1
0.1
0.01
0
6
12
18
24
30
Age (months)
B.
Controls
AMA1(3D7)
Age (months)
C.
Cases
MSP-2(Dd2)
Age (months)
D.
Controls
MSP-2(Dd2)
Age (months)
E.
Cases
MSP-3(3D7)
Age (months)
F.
Controls
MSP-3(3D7)
Age (months)
G.
Cases
MSP-1_19
Age (months)
H.
Controls
MSP-1-19
Age (months)
I.
Cases
PfRh2
Age (months)
J.
Controls
PfRh2
Age (months)

Supplementary Figure 2

Age-specific antibody titres, GIA levels and ADRB activity. IgG levels to AMA1 3D7 **(A)**, MSP-2 Dd2 **(B)**, MSP-3 3D7 **(C)**, MSP-1_{19} **(D)**, PfRh2 **(E)**, parasite schizont lysate **(F)** and the iRBC **(G)** in relation to infection status across different age-categories upto 2 years of age. A similar comparison of GIA levels **(H)** and ADRB activity **(I)** is also shown. Responses in parasite negative and parasite positive children are shown in grey and white; respectively. The solid horizontal lines on the box plots represent the medians and interquartile ranges. The horizontal dotted lines represent the levels from a pool of semi-immune adults resident in Kilifi. Parasite positivity was defined as presence of parasites confirmed either by microscopy or PCR. * $P<0.05$

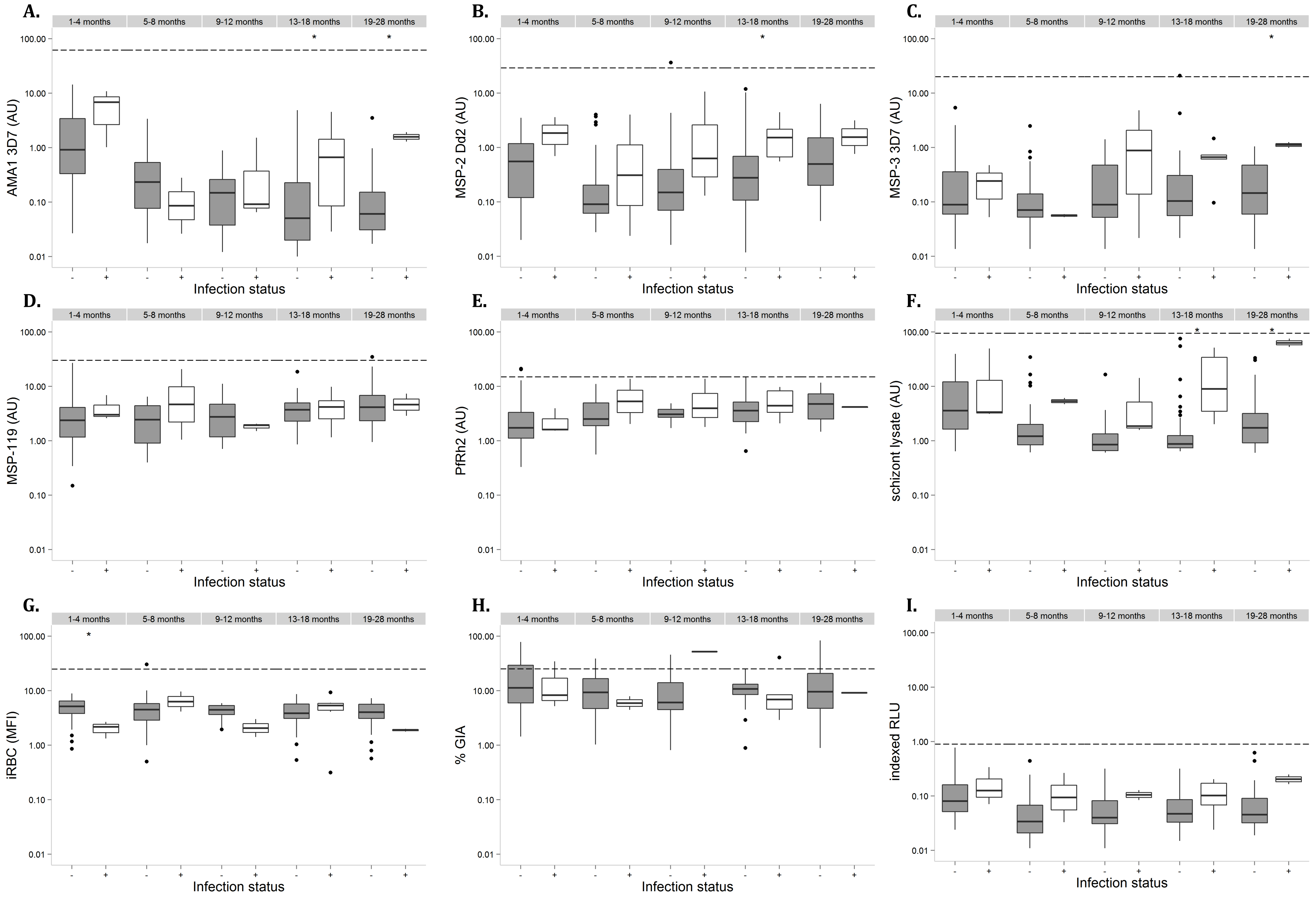
A.
B.
C.
D.
E.
F.
G.
H.
I.
1-4 months
5-8 months
9-12 months
13-18 months
19-28 months
100.00
10.00
1.00
0.10
0.01
AMA1 3D7 (AU)
MSP-2 Dd2 (AU)
MSP-3 3D7 (AU)
MSP-119 (AU)
PfRh2 (AU)
schizont lysate (AU)
iRBC (MFI)
% GIA
indexed RLU
Infection status
- +
*

Supplementary Figure 3

Plot matrix showing each individual's response against schizont extract, AMA1 (3D7), MSP-2 (Dd2), MSP-3 (3D7), MSP-1$_{19}$, *Pf*Rh2 and binding to the iRBC surface. Antibody concentrations were grouped according to the quartile distribution with 1, 2, 3 and 4 representing the 0 - 25th, 25th - 50th, 50th – 75th and 75th – 100th percentiles, respectively. Each row represents an individual while the columns show responses to each antigen according to the antibody concentration.

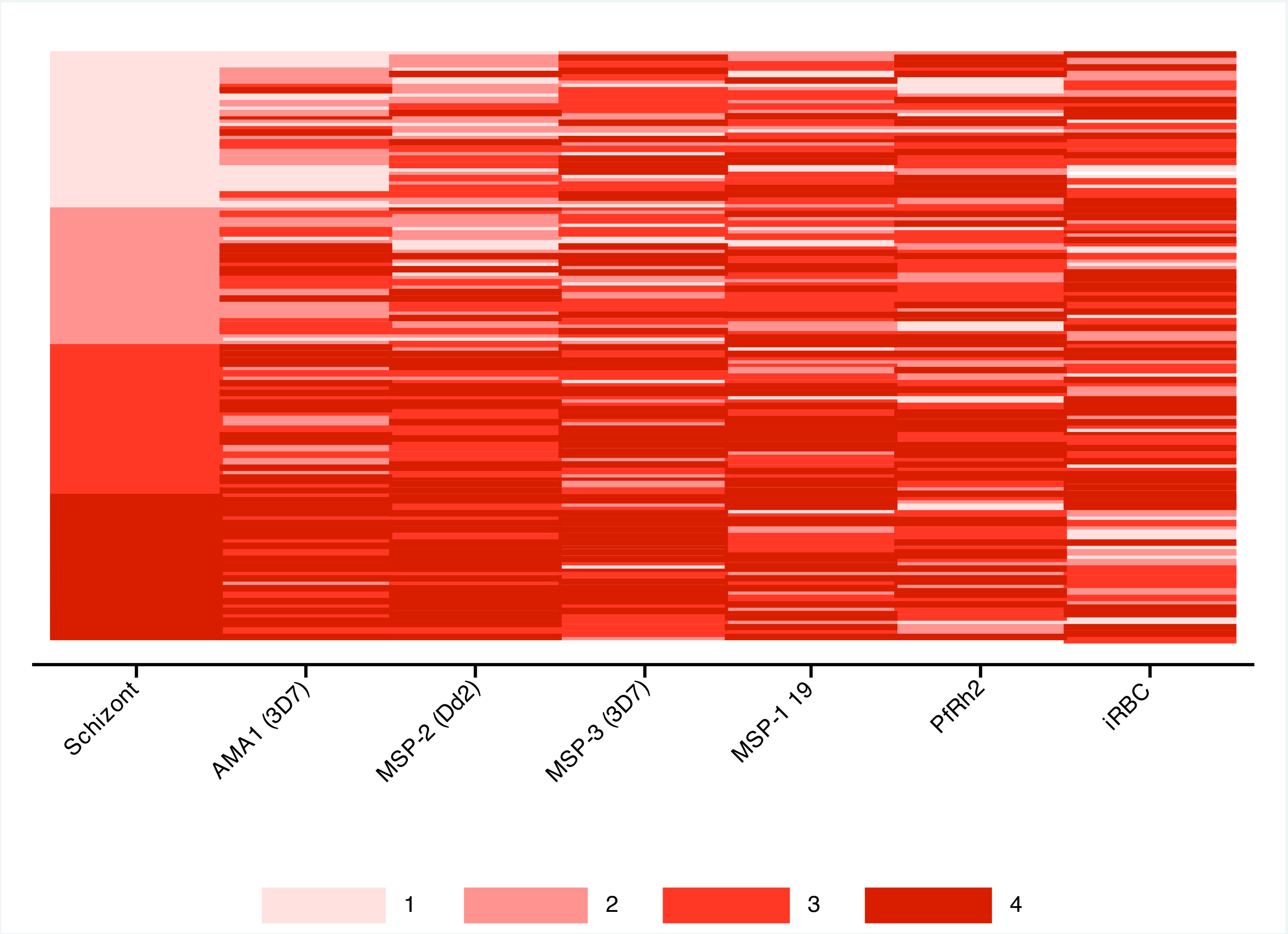

Supplementary Figure 4

Protective efficacy of anti-merozoite antibodies according to the number of antigens recognized. The odds ratios and 95% confidence intervals are indicated for responses to one, two, three and more than three antigens.

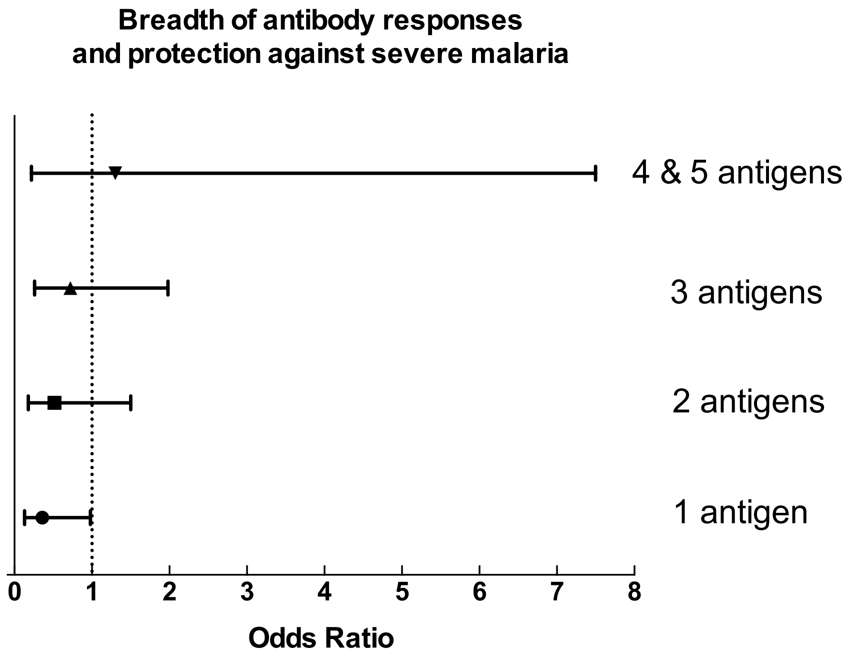

Supplementary Table 1

The association between antibody levels to specific merozoite antigens, the intact iRBC, GIA and ADRB activity and the odds of developing impaired consciousness.

	**Univariate analysis[a] OR (95% CI)**	**P-value**	**Multivariate analysis[b] OR (95% CI)**	**P-value**
**AMA1**	0.60 (0.17 – 2.01)	0.40	0.66 (0.14 – 2.95)	0.58
**MSP-2**	0.61 (0.19 – 1.95)	0.41	0.67 (0.17 – 2.57)	0.57
**MSP-3**	0.40 (0.11 – 1.40)	0.15	0.39 (0.09 – 1.66)	0.20
**MSP-1_{19}**	2.89 (0.28 – 29.55)	0.37	3.83 (0.33 – 43.68)	0.27
***Pf*Rh2**	0.70 (0.18 – 2.62)	0.60	0.70 (0.18 – 2.64)	0.61
**iRBC**	2.14 (0.67 – 6.79)	0.19	2.15 (0.67 – 6.87)	0.19
**GIA**	1.92 (0.67 – 5.46)	0.22	1.86 (0.65 – 5.34)	0.24
**ADRB**	0.41 (0.06 – 2.45)	0.33	0.46 (0.06 – 3.19)	0.43

[a]A conditional logistic regression model was used to calculate the odds of developing impaired consciousness during 2 years and 3 months of follow-up.

[b]Reactivity to *P. falciparum* schizont extract (fitted as a continuous covariate) was included in the model to account for differences in parasite exposure between those who developed impaired consciousness and those who developed severe anemia or respiratory distress.

Supplementary Table 2

The association between antibody levels to specific merozoite antigens, the intact iRBC, GIA and ADRB activity and the odds of developing severe malaria anemia.

	Univariate analysis[a] OR (95% CI)	P-value	Multivariate analysis[b] OR (95% CI)	P-value
**AMA1**	0.88 (0.23 – 3.31)	0.85	0.22 (0.02 – 1.69)	0.14
**MSP-2**	2.26 (0.68 – 7.52)	0.18	1.49 (0.37 – 5.97)	0.56
**MSP-3**	2.06 (0.59 – 7.18)	0.25	1.27 (0.29 – 5.56)	0.74
**MSP-1_{19}**	0.74 (0.07 – 7.69)	0.80	0.41 (0.03 – 5.16)	0.49
***Pf*Rh2**	0.82 (0.19 – 3.55)	0.79	0.81 (0.18 – 3.63)	0.78
**iRBC**	1.02 (0.29 – 3.56)	0.96	1.02 (0.28 – 3.68)	0.96
**GIA**	0.73 (0.23 – 2.27)	0.59	0.82 (0.25 – 2.61)	0.73
**ADRB**	0.42 (0.04 – 3.91)	0.44	0.13 (0.01 – 1.89)	0.13

[a]A conditional logistic regression model was used to calculate the odds of developing severe anemia during 2 years and 3 months of follow-up.

[b]Reactivity to *P. falciparum* schizont extract (fitted as a continuous covariate) was included in the model to account for differences in parasite exposure between those who developed severe anemia and those who developed impaired consciousness or respiratory distress.
